# Mixed‐matrix membranes incorporating hierarchical ZIF‐8 towards enhanced CO_2_/N_2_ separation

**DOI:** 10.1002/smo.20240066

**Published:** 2025-03-07

**Authors:** Ting Xia, Yuyang Wu, Taotao Ji, Wenjing Hu, Kunpeng Yu, Xinyu He, Ben Hang Yin, Yi Liu

**Affiliations:** ^1^ State Key Laboratory of Fine Chemicals School of Chemical Engineering Dalian University of Technology Dalian China; ^2^ Faculty of Engineering Robinson Research Institute Victoria University of Wellington Wellington New Zealand

**Keywords:** flue gas separation, hierarchical MOF, mixed‐matrix membranes, ZIF‐8 membrane

## Abstract

Metal‐organic framework (MOF) has been widely used as filler of mixed‐matrix membranes (MMMs) because of their tunable pore sizes, large surface areas, and rich functional groups. However, a relatively high diffusion barrier in the framework of bulk MOF fillers inevitably reduces gas permeability. Introduction of hierarchically porous structure represents an effective method for reducing guest diffusion resistance with no compromise in gas selectivity. In this study, hierarchical ZIF‐8 (H‐ZIF‐8) was prepared using carboxylated polystyrene (PS‐COOH) nanospheres as a hard template. Owing to the introduction of carboxyl groups, electrostatic interaction between PS nanospheres and Zn^2+^ ions is enhanced, facilitating uniform embedment of PS nanospheres in bulk ZIF‐8 filler. After dissolution of PS‐COOH nanospheres with dimethylformamide solvents, H‐ZIF‐8 with tunable textural properties is readily obtained. Gas permeation results indicate that compared with bulk ZIF‐8 filler, fast diffusion pathways for guest molecules are established in H‐ZIF‐8 filler, resulting in a CO_2_/N_2_ separation factor (SF) of 48.77 with CO_2_ permeability of 645.76 Barrer in terms of H‐ZIF‐8 MMMs with 6 wt % loading, which well exceeds the 2008 Robenson upper bound for CO_2_/N_2_ gas pair, thus showing promising prospects for high‐efficiency CO_2_ capture from flue gas.

## Graphical abstract

This work presents the synthesis of hierarchical ZIF‐8 (H‐ZIF‐8) using PS‐COOH nanospheres as the hard template. H‐ZIF‐8 MMMs with 6 wt % loading exhibited the CO_2_/N_2_ sepration factor and CO_2_ permeability 125% and 90%, respectively, than those of pure Pebax membrane.

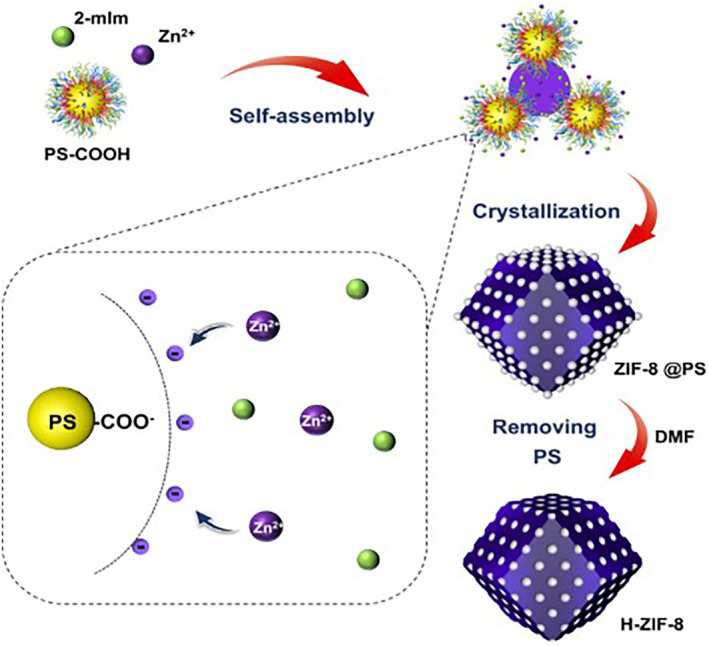

## 1 INTRODUCTION

Excessive CO_2_ emission poses a great threat to global warming, rendering it urgent to develop facile and economic protocols for high‐efficiency CO_2_ capture, utilization and storage (CCUS).[^1^, ^2^, ^3^] Among them, membrane technology shows obvious advantages in terms of high separation efficiency, low energy consumption, and small footprint.[^4^, ^5^] MMMs, which combine superior processability of polymeric phase and high efficiency of filler phase, have been demonstrated as a promising way for high‐efficiency CO_2_ capture.^[6]^ Polyether block amide (Pebax), consisting of flexible polyether (PE) segments and rigid polyamide (PA) segments, has high selectivity and permeability toward CO_2_. Different types of Pebax copolymers could be obtained by adjusting the ratio of PE segments. The permeability is positively associated with the amount of PE segments.^[7]^ Pebax 2533, containing 80% PE segments and 20% PA segments, shows the highest permeability compared with Pebax 1657, 1074,4033. Feng et al.^[8]^ synthesized Pebax 2533/SUM‐9 (a novel In(III)‐MOF with chelating hydroxamate ligands) MMMs. The MMMs with 1 wt % achieved a CO_2_/N_2_ selectivity of 24.69 with CO_2_ permeability of 539 Barrer.

Metal‐organic framework, a class of three‐dimensional porous materials consisting of inorganic metal nodes as secondary building units connected with organic ligands through chemical bonding,[^9^, ^10^, ^11^] possesses unique advantages such as tunable pore size, large surface areas, and rich functional groups.^[12]^ Among them, zeolitic imidazolate framework‐8 (ZIF‐8), an ordered crystalline porous material with sodalite (SOD) Polyvinylpyrrolidone zeolite structure consisting of zinc ions (Zn^2+^) and 2‐methylimidazole (2‐mIm) ligands,[^13^, ^14^] has been considered as superb candidate for membrane‐based separation.^[15]^ In general, N‐containing functional groups in MOF framework (such as imidazole ring and amino) can suppress the generation of interfacial defects and improve the affinity between MOF filler and polymer matrix through hydrogen bonding interaction between them[^16^, ^17^]; moreover, one peculiar structural feature of ZIF‐8 lies in its capability of structural transition through rotating (or swinging) 2‐mIm linkers under external stimuli,^[18]^ which could be used to manufacture MMMs adaptable to guest molecules with varying kinetic diameters,^[19]^ making it an ideal filler of high‐performance MMMs.

Previous studies indicated that direct use of bulk ZIF‐8 filler inevitably causes higher diffusion resistance.[^20^, ^21^, ^22^] To alleviate the negative effect of bulk ZIF‐8 filler on the gas permeability of MMMs, increasing the porosity of bulk ZIF‐8 filler has become indispensable. Creating hierarchically porous structures represents an effective method for providing a low‐resistance diffusion pathway of gas molecules.^[23]^ For instance, Su et al.^[24]^ synthesized a yolk–shell Au@ZIF‐8 nanoreactor through a sacrificial template (silica) strategy. The obtained Au@ZIF‐8 nanoreactor featured single‐crystalline ZIF‐8 shells with intrinsic micropores and introduced macropores, enabling selective catalysis of alcohols with molecular sizes larger than the aperture size of ZIF‐8, thus increasing the conversion rate of the reaction. He et al.^[25]^ synthesized polystyrene‐acrylate (PSA)‐modified hollow ZIF‐8 (PHZ) as filler of MMMs. Obtained PHZ‐2/Pebax MMMs (10 wt % loading) presented superior CO_2_/N_2_ SF of 87.9 and CO_2_ permeability of 172.4 Barrer. Li et al.^[26]^ developed a confined growth strategy to prepare 3DO sphere‐assembled ZIF‐8 single crystals and 3DO single‐crystalline ZIF‐8 sphere arrays by removing the 3DO macroporous polystyrene template. Nonetheless, it remains challenging for facile preparation of structurally intact hierarchical ZIF‐8 due to weak interactions between the MOF precursor and hard template as well as harsh post‐treatment steps (e.g., acidic or basic etching).

**FIGURE 1:**
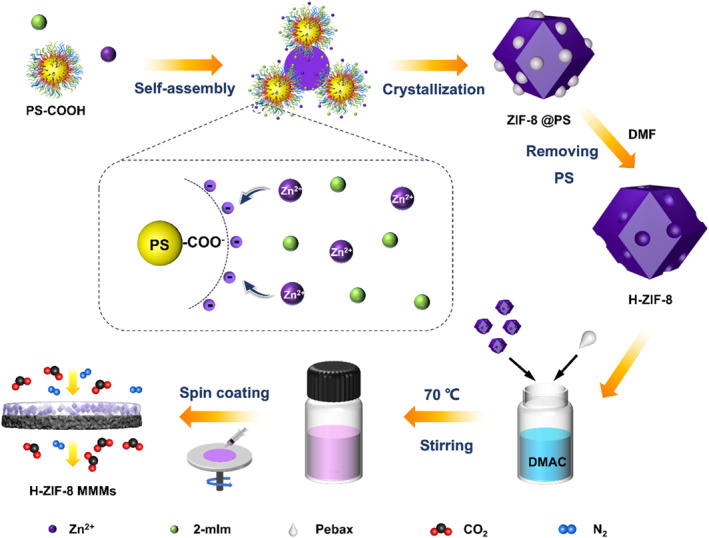
Schematic illustration of the preparation of H‐ZIF‐8 with polystyrene (PS‐COOH) nanospheres as the hard template and the formation process of H‐ZIF‐8 mixed‐matrix membranes (MMMs).

In this study, we prepared hierarchical ZIF‐8 (H‐ZIF‐8) using carboxylated polystyrene (PS‐COOH) nanospheres as hard templates (Figure 1). Since PS nanospheres are soluble in dimethylformamide (DMF), removal of hard templates under harsh acidic or alkaline conditions can be avoided. Moreover, our results showed that carboxylation of PS nanospheres prior to H‐ZIF‐8 synthesis was indispensable to enhance their electrostatic interaction with Zn^2+^ ions, thus leading to the formation of H‐ZIF‐8 with tunable textural properties. Subsequently, H‐ZIF‐8‐based MMMs were prepared through solution casting. Gas permeation results indicated that the obtained membrane exhibited CO_2_/N_2_ SF of 48.77 and CO_2_ permeability of 645.76 Barrer, which were 59% and 62% higher than those of bulk ZIF‐8 MMMs, thereby well exceeding the 2008 Robenson upper bound for CO_2_/N_2_ gas pair (Figure 1).

## 2 RESULTS AND DISCUSSION

### 2.1 Carboxylation of PS nanospheres

**FIGURE 2:**
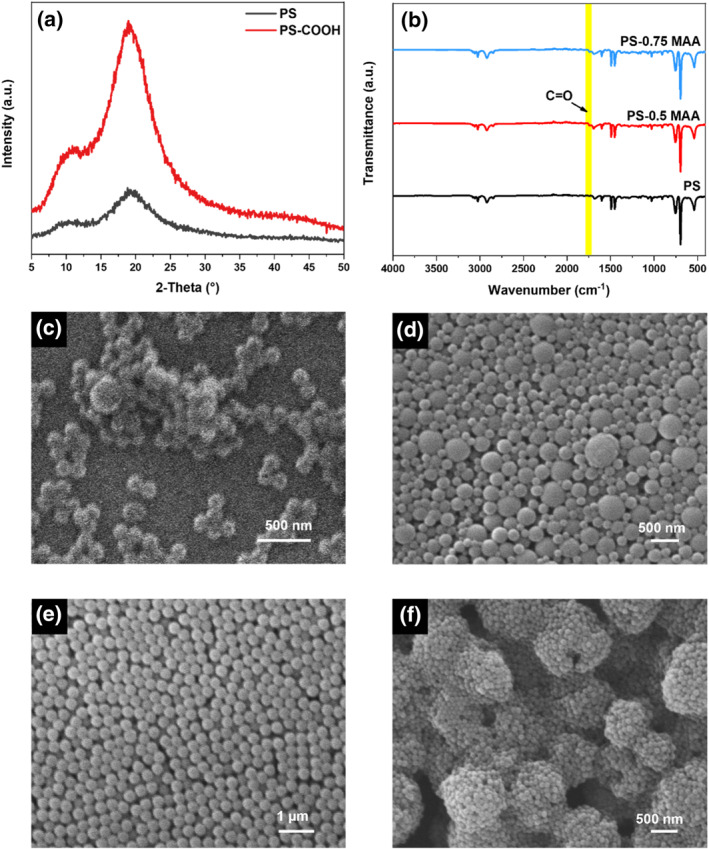
(a) X‐ray diffraction (XRD) patterns and (b) Fourier Transform Infrared (FT‐IR) spectra of PS and polystyrene (PS‐COOH) nanospheres. SEM images of PS‐COOH nanospheres with different addition amounts of polyvinylpyrrolidone (PVP) and MAA: (c) 1.0 g PVP/0.4 g α‐methacrylic acid (MAA), (d) 0.75 g PVP/0.4 g MAA, (e) 0.5 g PVP/0.4 g MAA and (f) 0.5 g PVP/0.5 g MAA.

Graft modification represents a facile method for surface modification of PS nanospheres. Styrene monomers could be polymerized with double bond‐containing molecules.^[27]^ Therefore, PS‐COOH nanoparticles could be obtained through adding a given amount of α‐methacrylic acid (MAA) during the reaction. X‐ray diffraction (XRD) patterns (Figure 2A) showed two broad diffraction peaks located between 10° and 19°, which was consistent with that of PS nanospheres as reported in the literature.^[28]^ Relevant Fourier Transform Infrared (FT‐IR) spectrum (Figure 2B) manifested a vibrational peak located at 1700 m^−1^, indicating successful anchoring of ‐COOH functional groups on PS nanospheres.

Our results revealed that the dosage of MAA and polyvinylpyrrolidone (PVP) exerted significant influence on the morphology and size of PS‐COOH spheres. According to the literature,^[29]^ PVP micelles adsorbed on the surface of primary particles could enhance the stability of reaction system, resulting in the formation of PS spheres with uniform size and high sphericity. However, adding excessive amount of PVP would lead to the formation of negatively charged primary particles, resulting in their repulsive interaction with MAA. Therefore, increasing PVP amount from 0.5 to 1 g led to gradually weakened polymerization effect, resulting in reduced morphological regularity (Figure 2C) and size uniformity (Figure 2D) of PS‐COOH nanospheres. Under optimized reaction conditions, obtained PS‐COOH nanoparticles possessed uniform size (~200 nm), high sphericity, and excellent monodispersity (Figure 2E), which was beneficial for creating uniform macropores in bulk ZIF‐8 crystals. Simultaneously, with increasing MAA concentration, particle agglomeration became more serious (Figure 2F).

### 2.2 Preparation of the H‐ZIF‐8 filler

**FIGURE 3:**
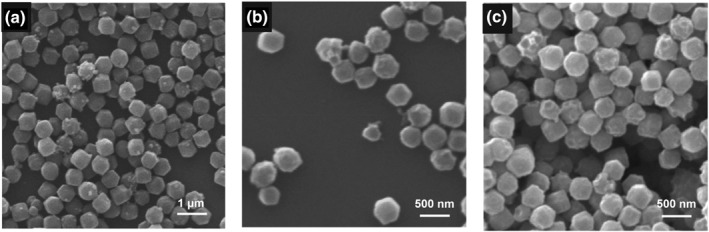
SEM images of ZIF‐8 crystals synthesized with different addition amount of PS nanospheres: (a) 0 g ml^−1^, (b) 0.1 g ml^−1^ and (c) 0.2 g ml^−1^ in the precursor solution.

The H‐ZIF‐8 filler could be facilely prepared with PS‐COOH nanospheres as the hard template. To gain insights into the formation of hierarchically porous structures, blank experiments were conducted to prepare ZIF‐8 in the absence and presence of PS nanospheres as hard templates. Initially, bulk ZIF‐8 nanocrystals (B‐ZIF‐8) were prepared in the absence of hard template. SEM results (Figure 3A) indicated that obtained B‐ZIF‐8 were dodecahedral‐shaped with average size of ∼570 nm; moreover, hierarchical pores were absent in crystals. Subsequently, PS nanospheres were added to ZIF‐8 precursor solution. Nonetheless, owing to the weak interaction between ZIF‐8 precursor solution and PS nanospheres, it remained difficult to trigger heterogeneous nucleation of ZIF‐8 on PS nanospheres, resulting in the generation of substantial B‐ZIF‐8 in bulk solution (Figure 3B). Even upon increasing the concentration of PS nanospheres to 0.2 g ml^−1^, hierarchically porous ZIF‐8 crystals still could not be obtained (Figure 3C).

**FIGURE 4:**
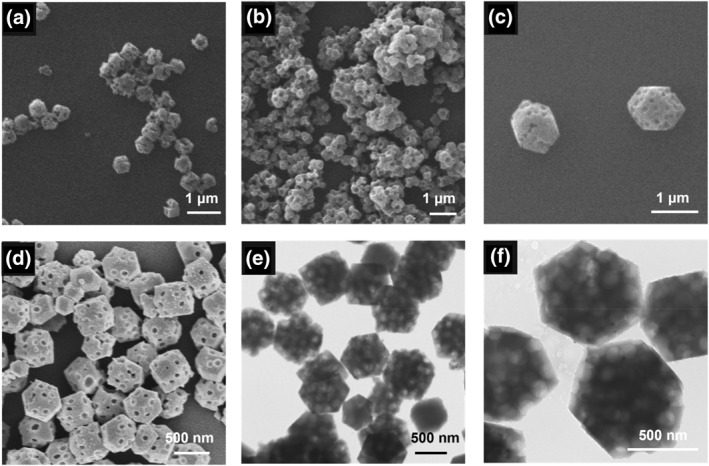
SEM images of H‐ZIF‐8 prepared with polystyrene (PS‐COOH) nanospheres containing different amounts of MAA: (a) 0.7 g α‐methacrylic acid (MAA), (b) 0.6 g MAA, (c) 0.5 g MAA and (d) 0.4 g MAA. Transmission electron microscopy (TEM) images of H‐ZIF‐8 prepared with PS‐COOH nanospheres containing different amounts of MAA: (e) 0.5 g MAA, (f) 0.4 g MAA.

It should be noted that compared with PS nanospheres, electrostatic interaction between carboxyl groups on the PS‐COOH surface and Zn^2+^ ions in the bulk solution could enhance the affinity between them, facilitating heterogenous nucleation of ZIF‐8 on the surface of PS‐COOH nanospheres. As shown in Figure 4, hierarchical pores were successfully introduced in bulk ZIF‐8 crystals with PS‐COOH nanospheres as the templating agent. It should be noted that the number of carboxyl groups on the PS‐COOH nanospheres exerted a remarkable effect on the morphology of H‐ZIF‐8. Upon adding > 0.5 g of MAA in the precursor solution, the obtained H‐ZIF‐8 exhibited poor morphology and severe agglomeration (Figure 4A–B). This was rational, since along with excessive addition of MAA, severe agglomeration between PS‐COOH nanospheres would occur, which negatively affected the uniformity and monodispersity of H‐ZIF‐8; in addition, the lone pair of electrons of N in 2‐mIm ligands had certain alkalinity, which was undesired for precise control nucleation and growth kinetics of ZIF‐8 in carboxylic acid‐containing solutions. Therefore, the addition amount of MAA should be no higher than 0.5 g in order to maintain uniform spheric‐shaped macropore size and regular dodecahedral morphology of H‐ZIF‐8. In addition, our results indicated that the size of H‐ZIF‐8 was positively associated with the amount of MAA. For instance, the size of H‐ZIF‐8 gradually increased from 600 to 960 nm upon increasing the MAA amount from 0.4 to 0.5 g (Figure 4C–F).

**FIGURE 5:**
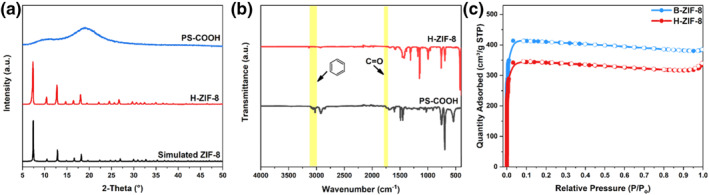
(a) X‐ray diffraction (XRD) patterns, (b) Fourier Transform Infrared (FT‐IR) spectra of H‐ZIF‐8 and polystyrene (PS‐COOH) nanospheres, and (c) N_2_ adsorption/desorption isotherms of H‐ZIF‐8 and B‐ZIF‐8.

Relevant XRD patterns (Figure 5A) further showed that the obtained H‐ZIF‐8 belonged to the pure ZIF‐8 phase; simultaneously, the diffraction peak of PS‐COOH located at 10−25° completely disappeared, indicating that the PS‐COOH template had been completely removed after being treated with DMF; further, FT‐IR spectra (Figure 5B) revealed that the absorption peaks derived from PS‐COOH were absent, confirming that the PS‐COOH nanospheres had been completely removed. The textural properties of H‐ZIF‐8 were further studied by measuring N_2_ sorption isotherms at 77 K (Figure 5C). H‐ZIF‐8 exhibited type‐I isotherm and its Brunauer‐Emmet‐Teller (BET) surface areas reached 1017.70 m^2^ g^−1^, which was slightly lower than that of B‐ZIF‐8 (1220.21 m^2^ g^−1^). This was rational since some micropores were simultaneously removed, accompanying with the introduction of macropores in H‐ZIF‐8.

### 2.3 Preparation of H‐ZIF‐8 mixed‐matrix membranes

**FIGURE 6:**
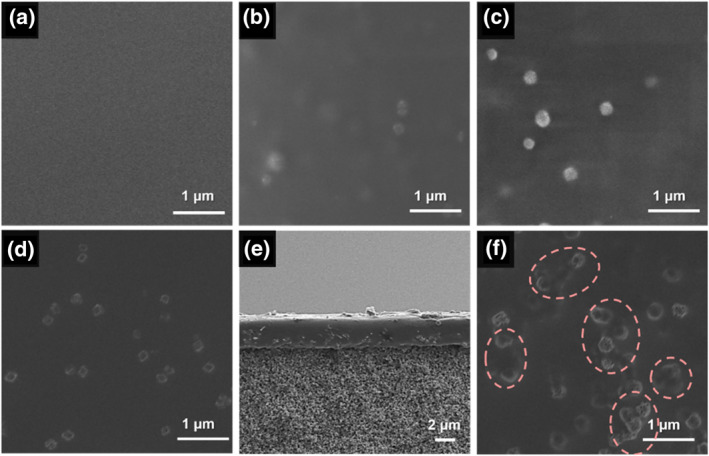
SEM images of (a) pure Pebax polymer membrane, (b) H‐ZIF‐M −2 wt %, (c) H‐ZIF‐M‐4 wt %, (d), (e) H‐ZIF‐M‐6 wt % and (f) H‐ZIF‐M‐8 wt %.

In the next step, H‐ZIF‐8/Pebax 2533 MMMs (H‐ZIF‐M) with different H‐ZIF‐8 loadings were prepared using the solution casting method. The area of prepared H‐ZIF‐8/Pebax 2533 MMMs was 2.5 cm^2^. As shown in Figure 6A–D, the surface of obtained H‐ZIF‐M appeared uniform and continuous with no visible defects at H‐ZIF‐8 loading no higher than 6 wt %. Cross‐sectional SEM images (Supporting Information S1) further indicated that the thicknesses of H‐ZIF‐M increased with increasing doping amount of H‐ZIF‐8 fillers at H‐ZIF‐8 loading no higher than 6 wt %. Moreover, the thickness of H‐ZIF‐M‐6 wt % reached 3.2 μm (Supporting Information S1) with no severe infiltration of the polymer matrix into the substrate pores (Figure 6E). However, severe agglomeration occurred upon further increasing filler loading to 8 wt % (Figure 6F), which inevitably led to the generation of interfacial defects within the membrane.

### 2.4 Gas permeation test

**FIGURE 7:**
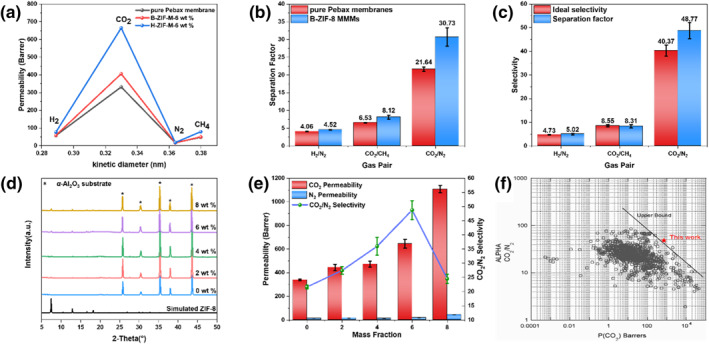
(a) Gas permeation properties of pure Pebax‐2533 membrane, B‐ZIF‐M‐6 wt % and H‐ZIF‐M‐6 wt %. (b) separation factor (SF) of equimolar H_2_/N_2_, CO_2_/CH_4_, and CO_2_/N_2_ mixture on B‐ZIF‐M‐6 wt % and pure Pebax membranes. (c) Ideal selectivity and SF of equimolar H_2_/N_2_, CO_2_/CH_4_, and CO_2_/N_2_ mixture on H‐ZIF‐M‐6 wt %. (d) X‐ray diffraction (XRD) patterns of mixed‐matrix membranes (MMMs) with different H‐ZIF‐8 loadings. (e) CO_2_/N_2_ separation performances of H‐ZIF‐8 MMMs with different loadings. (f) Comparison with the CO_2_/N_2_ separation performance of H‐ZIF‐M‐6 wt % with the Robeson 2008 upper bound for CO_2_/N_2_ gas pair. Reproduced with permission.^[31]^ Copyright 2008, Elsevier.

Finally, gas permeation tests were performed using the Wicke–Kallen technique under ambient conditions. Volumetric flow rates of single H_2_, CO_2_, N_2_ and CH_4_ through H‐ZIF‐M‐6 wt %, B‐ZIF‐M‐6 wt % and pure Pebax 2533 membranes were first evaluated. As shown in Figure 7A, the single gas permeation behavior in all the above membranes was similar. Among them, CO_2_ exhibited the highest permeability. More detailed comparison of CO_2_ permeability in H‐ZIF‐M‐6 wt %, B‐ZIF‐M‐6 wt % and pure Pebax 2533 membranes implied that CO_2_ permeability of H‐ZIF‐M‐6 wt % (664.09 Barrer) was improved by 101% over pure Pebax 2533 membrane (330.76 Barrer) and 64% over B‐ZIF‐M‐6 wt % (405.23 Barrer). Obviously, introduction of hierarchy in ZIF‐8 framework significantly reduced the diffusion barrier, thereby contributing to faster permeation of CO_2_ in the membrane.

**FIGURE 8:**
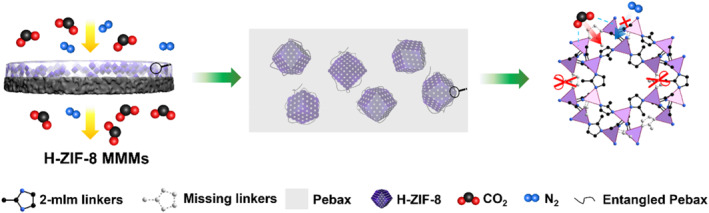
Schematic illustration of the gas separation mechanism of H‐ZIF‐8 mixed‐matrix membranes (MMMs). Color code: black = C; red = O; blue = N. H atoms are omitted for concision.

We further evaluated the separation performance of the equimolar CO_2_/N_2_ gas mixture on H‐ZIF‐M‐6 wt %, B‐ZIF‐M‐6 wt % and pure Pebax 2533 membranes (Figure 7B,C). Among them, CO_2_/N_2_ SF and CO_2_ permeability of pure Pebax membrane reached 21.64 and 339.42 Barrer, respectively; while those of B‐ZIF‐M‐6 wt % reached 30.73 and 398.77 Barrer, respectively, which were 42% and 17% higher than those of pure Pebax membrane. Obviously, the imidazole ring in 2‐mIm suppressed the generation of interfacial defects by improving the affinity between the ZIF‐8 and polymer. Reduced interfactional defects and sieving effects worked together to enhance the performance of CO_2_ separation. In terms of H‐ZIF‐M‐6 wt %, CO_2_/N_2_ SF further increased to 48.77, which was 59% higher than that of B‐ZIF‐M‐6 wt % and 125% higher than that of pure Pebax membrane. Simultaneously, corresponding XRD patterns revealed that diffraction peaks located at 2θ values of 7.4°, 10.5°, 12.8°, 16.6° and 18.3°, which were assignable to the pure ZIF‐8 phase, appeared in the obtained MMMs; simultaneously, the intensity of the above diffraction peaks increased with increasing doping amount of H‐ZIF‐8 fillers in the polymer matrix (Figure 7D). The effect of filler loading on CO_2_/N_2_ selectivity and CO_2_ permeability of the obtained MMMs was investigated. As shown in Figure 7E, when the H‐ZIF‐8 loading was lower than 6 wt %, both CO_2_ and N_2_ permeability as well as CO_2_/N_2_ SF increased with increasing H‐ZIF‐8 loading; nonetheless, further increasing H‐ZIF‐8 loading to 8 wt % led to a more pronounced increase of N_2_ permeability and therefore rapid decay in CO_2_/N_2_ SF from 48.77 to 24.58. This could be attributed to severe agglomeration of H‐ZIF‐8 in the membrane which led to the generation of interfacial defects between H‐ZIF‐8 filler and polymer matrix, thereby negatively affecting the CO_2_/N_2_ SF of obtained MMMs. We speculated that the introduction of PS‐COOH nanoparticles during the nucleation and crystallization of H‐ZIF‐8 and DMF etching of PS‐COOH nanoparticles would lead to more open metal sites in ZIF‐8 framework. As shown in Figure 8, open metal sites could form π‐bond interactions with CO_2_ molecules, resulting in enhanced affinity interactions between H‐ZIF‐8 and CO_2_, and therefore, increased CO_2_/N_2_ SF^[30]^; moreover, owing to the introduction of a hierarchically porous structure, the CO_2_ permeability increased to 645.76 Barrer, representing 62% increase compared with B‐ZIF‐M‐6 wt% and a 90% increase compared with pure Pebax membrane, surpassing the 2008 Robeson upper bound for CO_2_/N_2_ gas pair (Figure 7F).

As shown in Figure 7E, when the H‐ZIF‐8 loading was lower than 6 wt %, both CO_2_ and N_2_ permeability as well as CO_2_/N_2_ SF increased with increasing H‐ZIF‐8 loading; nonetheless, further increasing H‐ZIF‐8 loading to 8 wt % led to a more pronounced increase of N_2_ permeability and therefore rapid decay in CO_2_/N_2_ SF from 48.77 to 24.58. This could be attributed to severe agglomeration of H‐ZIF‐8 in the membrane which led to the generation of interfacial defects between H‐ZIF‐8 filler and polymer matrix, thereby negatively affecting the CO_2_/N_2_ SF of obtained MMMs.

## 3 CONCLUSION

In this study, we facilely synthesized H‐ZIF‐8 using PS‐COOH nanospheres as a hard template. The electrostatic affinity between PS‐COOH nanospheres and ZIF‐8 precursor was found to be the key construct hierarchically porous structure and inhibition of ZIF‐8 formation in precursor solution. The introduction of uniform macropores in bulk ZIF‐8 crystals provided not only rapid diffusion channels for guest molecules but also higher affinity interaction between H‐ZIF‐8 filler and CO_2_ by forming more open metal sites. H‐ZIF‐8 MMMs with 6 wt % loading exhibited a CO_2_/N_2_ SF of 48.77 and CO_2_ permeability of 645.76 Barrer, which well exceeded the 2008 Robenson upper bound for CO_2_/N_2_ gas pair, thus showing great promise in practical CO_2_ capture from flue gas.

## 4 EXPERIMENTAL SECTION/METHODS

### 4.1 Reagents and materials

Styrene (99%, Macklin), sodium hydroxide pellets (NaOH, 96%, Macklin), potassium persulfate (K_2_S_2_O_8_, 99%, Macklin), MAA(C_4_H_6_O_2_, 99%, Macklin), (PVP, Mw = 58,000, 99%, Macklin), zinc nitrate hexahydrate (Zn(NO_3_)_2_·6H_2_O, 99%, Aladdin), 2‐methylimidazole (C_4_H_6_N, 98%, Macklin), Pebax 2533 [containing 80 wt % poly(tetramethylene oxide)(PTMO), 20 wt % polyamide‐12 (PA‐12), Arkema], methanol (CH_4_O, 99.5%, Tianjin Kemiou), N,N‐dimethylformamide (DMF, C_3_H_7_NO, 99.8%, Tianjin Kemiou), N,N‐dimethylacetamide (DMAc, C_4_H_9_NO, 99%, Macklin), and ethanol (C_2_H_6_O,99.7%, Tianjin Kemiou) were used as received without further purification. Porous α‐Al_2_O_3_ disks with pore size of 70 nm, diameter of 18 mm, and thickness of 1 mm were purchased from Fraunhofer IKTS, Germany.

### 4.2 Preparation of PS‐COOH nanospheres

Monodispersed PS‐COOH nanospheres were synthesized according to previous literature with slight modification.^[28]^ Briefly, styrene (13 g) was washed with 10 wt % NaOH solution (5 ml) and deionized (DI) water to remove stabilizer from the styrene monomer. Subsequently, washed styrene and MAA (0.4 g) were added to a round‐bottom flask, followed by the addition of an aqueous solution (115 ml) containing PVP (0.5 g). The mixture was fluxed at 95°C under magnetic stirring for 30 min. In the next step, an aqueous solution (15 ml) containing K_2_S_2_O_8_ (0.1 g) was added to the flask and stirred at 95°C for 24 h. The obtained product was sequentially centrifuged, washed with DI water, and finally dried at 50°C overnight. The PVP/MAA ratio was varied to investigate its effect on the morphology of PS‐COOH nanospheres.

### 4.3 Preparation of H‐ZIF‐8

Zn(NO_3_)_2_·6H_2_O (0.815 g) and PS‐COOH nanospheres (0.5 g) were added to methanol (12 ml). The obtained suspension was named as solution A. 2‐mIm (0.675 g) was added to methanol (12 ml). The above solution was named as solution B. In the next step, solution B was added to solution A, followed by reaction at room temperature under stirring for 24 h. The obtained powders were centrifuged, washed with ethanol, and dried at 70°C. Dried products were immersed in DMF for 5 days to remove PS‐COOH, washed repeatedly with ethanol, and finally dried overnight at 70°C before further use.

### 4.4 Synthesis of B‐ZIF‐8 Crystals and P‐ZIF‐8 crystals

For comparison, bulk ZIF‐8 crystals (denoted as B‐ZIF‐8) were synthesized with a similar method except in the absence of PS‐COOH as hard template. ZIF‐8 crystals synthesized with a similar method except using PS nanospheres as hard template.

### 4.5 Fabrication of ZIF‐8 MMMs

A series of H‐ZIF‐8 MMMs with different loadings were prepared. H‐ZIF‐8 filler loading was defined as the following equation:

(1) $$H‐\text{ZIF}‐8\;\text{loading}\;\left(\text{wt}\;\%\right)=\frac{m_{H‐\text{ZIF}‐8}}{m_{\text{Pebax}\;}+{\;m}_{H‐\text{ZIF}‐8}}\times 100\%$$

Specific experimental steps were described as MMMs with 6 wt % H‐ZIF‐8 filler loading. H‐ZIF‐8 (0.016 g) was added to DMAc (4.75 g) to obtain a homogeneous H‐ZIF‐8 suspension. Subsequently, Pebax 2533 particles (0.25 g) were added to the solution under stirring at 70°C for 2 h. Finally, the mixed solution was spin‐coated on porous α‐Al_2_O_3_ disk at 3000 rpm for 60 s and dried overnight at 70°C. H‐ZIF‐8 MMMs were denoted as H‐ZIF‐M‐x (x represented the loading of filler). B‐ZIF‐8 MMMs were prepared in the same way as H‐ZIF‐8 MMMs except that B‐ZIF‐8 was used as filler. The obtained samples were denoted as B‐ZIF‐M‐x.

### 4.6 Characterization

X‐ray diffraction patterns were collected on a Rigaku SmartLab diffractometer with Cu Kα radiation (*λ* = 0.15418 nm) at 45 kV and 200 mA. Scanning electron microscopy (SEM) images were evaluated with a FlexSEM 1000 instrument (Hitachi Co.) at accelerating voltages of 10 and 15 kV. Transmission electron microscopy images were evaluated by JEM2100 Instruments (JEOL, Japan) at an operation voltage of 200 kV. Physical adsorption analysis was performed on a Mike ASAP 2020 Plus analyzer. Fourier Transform Infrared analysis was performed on an EQUINOX55 infrared spectrometer (Bruker, Germany).

### 4.7 Gas permeation test

H‐ZIF‐8 and B‐ZIF‐8 MMMs were fixed in a membrane module sealed with O‐rings. Volumetric flow rates of the feed (single gases or binary gas mixtures) were kept at 50 ml min^−1^, and permeated gas was removed from the permeate side by sweep gas (He). The pressure difference in both feed and permeate sides was kept at 1 bar. A calibrated gas chromatograph (7890B, Agilent) was employed to measure the concentration of each gas on the permeate side. Separation factor *α*_A/B_ was calculated from the quotient of the molar fractions of the components (A and B) in the feed and permeate sides using the following equation:

(2) $$\alpha_{A/B}=\frac{X_{A,\text{perm}}/X_{B,\text{perm}}}{X_{A,\text{feed}}/X_{B,\text{feed}}}$$

Ideal selectivity, that is, *α*_A/B_ (ideal), was defined as the single gas permeance ratio of gases A and B:

(3) $$\alpha_{A/B}\left(\text{ideal}\right)=\frac{P_{A}\left(\text{permeance}\right)}{P_{B}\left(\text{permeance}\right)}$$

The gas permeability (*P*_*i*_, mol m^−1^ s^−1^ Pa^−1^) was expressed as the flux of component *i* per unit area (*J*_*i*_, mol m^−2^ s^−1^) multiplied by the effective thickness of the MOF membrane (*L*, m) and divided by its driving force, that is, partial pressure differences (Δ*P*_*i*_, Pa) between the upstream and downstream sides through the membrane:

(4) $${\;P}_{i}\left(\text{permeability}\right)=P_{i}\left(\text{permeance}\right)\times L=\frac{J_{i}L}{∆P_{i}}$$

## CONFLICT OF INTEREST STATEMENT

The authors declare no conflicts of interest.

## ETHICS STATEMENT

No animal or human experiments were involved in this study.

## Supporting information

Supporting Information S1

## Data Availability

The data that support the findings of this study are available from the corresponding author upon reasonable request.
