# Supplementary material for: Mixed‐matrix membranes incorporating hierarchical ZIF‐8 towards enhanced CO2/N2 separation
Source: Smart Mol. 2025 Mar 7;3(2):e20240066. doi: 10.1002/smo.20240066 (PMC12262010; doi:10.1002/smo.20240066)
Supplement: Supplementary file 1 — Supporting Information S1 [file SMO2-3-e20240066-s001.docx]

Supporting Information

**Mixed-Matrix Membranes Incorporating Hierarchical ZIF-8 towards Enhanced CO_2_/N_2_ Separation**

Ting Xia, Yuyang Wu, Taotao Ji, Wenjing Hu, Kunpeng Yu, Xinyu He, Ben Hang Yin, and Yi Liu*

**
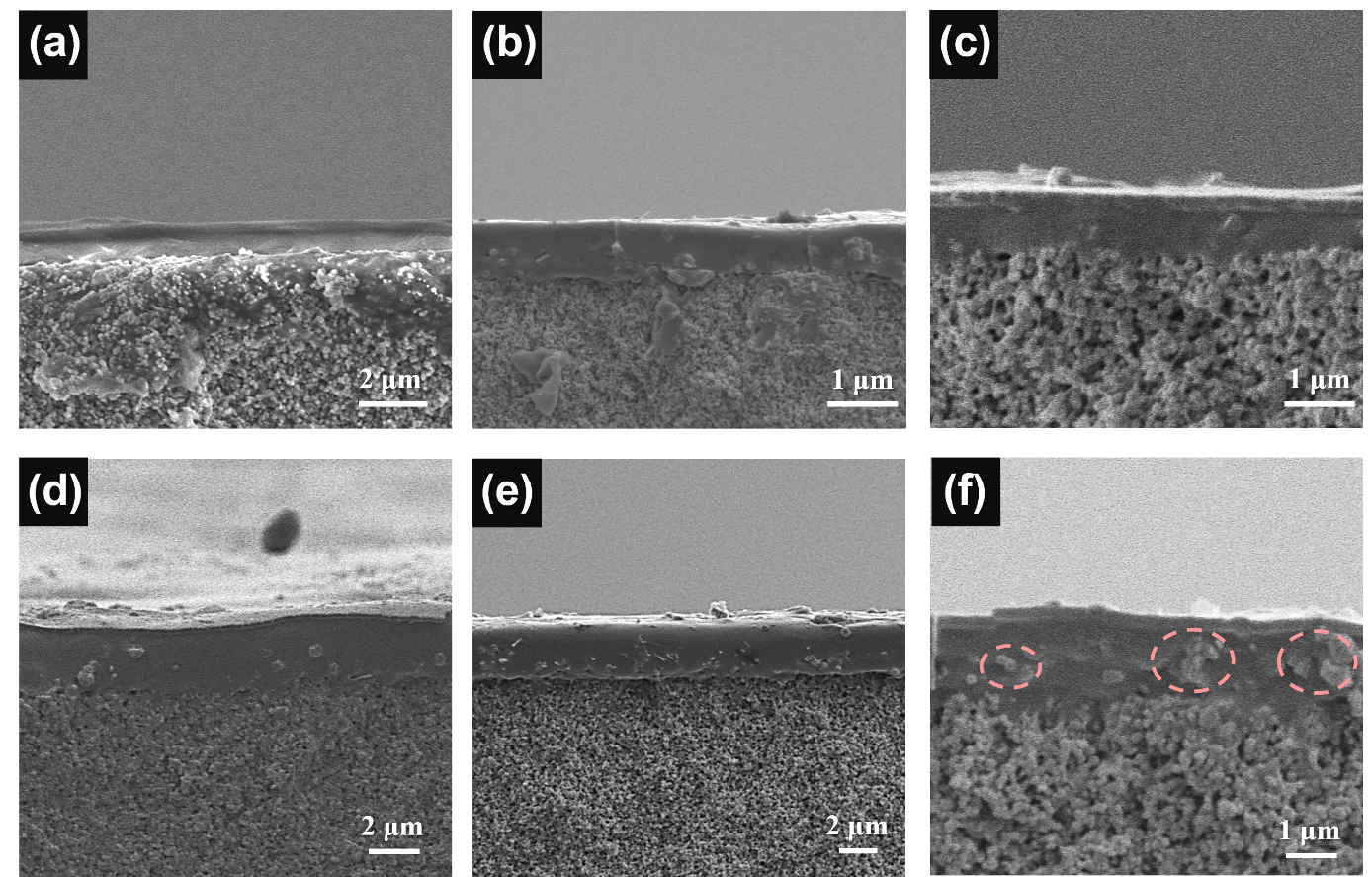
**

**Figure S1.** Cross-sectional SEM image of (a) Pebax 2533, (b) B-ZIF-M-6 wt %, (c) H-ZIF-M-2 wt %, (d) H-ZIF-M-4 wt %, (e) H-ZIF-M-6 wt %, and (f) H-ZIF-M-8 wt %.

**Table S1.** The thicknesses of MMMs with different loading.

| Sample | Thicknesses  (μm) |
| --- | --- |
| Pebax 2533 | 1.3 |
| B-ZIF-M-6 wt % | 2.8 |
| H-ZIF-M-2 wt % | 0.9 |
| H-ZIF-M-4 wt % | 2.8 |
| H-ZIF-M-6 wt % | 3.2 |
| H-ZIF-M-8 wt % | 1.9 |
